# Evolution of movement rate increases the effectiveness of marine reserves for the conservation of pelagic fishes

**DOI:** 10.1111/eva.12460

**Published:** 2017-03-15

**Authors:** Jonathan A. Mee, Sarah P. Otto, Daniel Pauly

**Affiliations:** ^1^ Department of BiologyMount Royal UniversityCalgaryABCanada; ^2^ Biodiversity Research CentreUniversity of British ColumbiaVancouverBCCanada; ^3^ Sea Around Us, Institute for Oceans and FisheriesUniversity of British ColumbiaVancouverBCCanada

**Keywords:** fisheries‐induced evolution, marine protected areas, migration and dispersal, pelagic fishes, philopatry

## Abstract

Current debates about the efficacy of no‐take marine reserves (MR) in protecting large pelagic fish such as tuna and sharks have usually not considered the evolutionary dimension of this issue, which emerges because the propensity to swim away from a given place, like any other biological trait, will probably vary in a heritable fashion among individuals. Here, based on spatially explicit simulations, we investigated whether selection to remain in MRs to avoid higher fishing mortality can lead to the evolution of more philopatric fish. Our simulations, which covered a range of life histories among tuna species (skipjack tuna vs. Atlantic bluefin tuna) and shark species (great white sharks vs. spiny dogfish), suggested that MRs were most effective at maintaining viable population sizes when movement distances were lowest. Decreased movement rate evolved following the establishment of marine reserves, and this evolution occurred more rapidly with higher fishing pressure. Evolutionary reductions in movement rate led to increases in within‐reserve population sizes over the course of the 50 years following MR establishment, although this varied among life histories, with skipjack responding fastest and great white sharks slowest. Our results suggest the evolution of decreased movement can augment the efficacy of marine reserves, especially for species, such as skipjack tuna, with relatively short generation times. Even when movement rates did not evolve substantially over 50 years (e.g., given long generation times or little heritable variation), marine reserves were an effective tool for the conservation of fish populations when mean movement rates were low or MRs were large.

## 1 Introduction

Given the generalized fisheries‐induced declines of marine biodiversity in the last decades (Watson et al., 2013), there is, among marine biologists, a wide agreement that marine protected areas (MPAs) and especially no‐take marine reserves (MRs) are needed to avoid further biodiversity losses (Lubchenco, Palumbi, Gaines, & Andelman, 2003; Pauly et al., 2002; Wood, Fish, Laughren, & Pauly, 2008) and to regain (at least partly) what has been lost (Roberts, Hawkins, & Gell, 2005). However, there is a concern that marine protected areas will have a negligible effect on preserving stocks of pelagic fishes in the Pacific Ocean (e.g., Sibert, Senina, Lehodey, & Hampton, 2012). Notably, tuna and sharks move great distances, and most marine reserves are relatively small. Many tuna and sharks can travel 1,000s of nautical miles in a single year (Bonfil et al., 2005; Sibert & Hampton, 2003), and reserves may afford protection only for a limited portion of a species’ life cycle, especially for migratory species (McAllister, Barnett, Lyle, & Semmens, 2015). Yet, even within such species, variation exists, with many individuals displacing less than 50 nautical miles (NM) between tagging and recapture, and some displacing more than 1,000 NM (Sibert & Hampton, 2003). Whereas the effect of marine reserves on evolution of size at maturation has received considerable attention (Baskett, Levin, Gaines, & Dushoff, 2005; Dunlop, Baskett, Heino, & Dieckmann, 2009; Miethe, Pitchford, & Dytham, 2009), a previously neglected effect of marine reserves (but see Miethe et al., 2009) is that more mobile individuals are more likely to leave a reserve and be fished, while less mobile (or more philopatric) individuals can remain within (or return to) reserves and survive. As a result, over time, the number of fish exhibiting low movement rates should rise. An evolutionary change in movement rate may, in turn, result in an increased density of fish in the marine reserves. There is a growing recognition that fisheries management and conservation should account for evolutionary effects (Fraser, 2013; Palkovacs, 2011). By neglecting the potential evolution of reduced movement rate following reserve establishment, managers may underestimate the efficacy of marine reserves.

For a trait to evolve, it must be heritable. Estimates of heritability for movement rate for pelagic species such as tuna and sharks do not, however, currently exist. There is a large amount of variation among individuals in movement rate (Sibert & Hampton, 2003), and it is unlikely that none of the variation in movement rate is heritable. Furthermore, there is an extensive literature demonstrating the heritability (and genetic basis) of movement rate across taxa. For example, in one of the best‐studied cases, a large amount of the variation in movement rate of larval and adult fruit flies depends on the genotype at the *for* (“foraging”) locus (Edelsparre, Vesterberg, Lim, Anwari, & Fitzpatrick, 2014; Sokolowski, 1980). A heritable genetic basis for many behaviors related to movement rate has also been identified in a variety of fish species (Alos, Palmer, & Arlinghaus, 2012; Booth, Hairston, & Flecker, 2013; Breau, Weir, & Grant, 2007; Conrad, Weinersmith, Brodin, Saltz, & Sih, 2011; Coombs & Rodriguez, 2007; Diamond et al., 2007; Laine, Primmer, Herczeg, Merila, & Shikano, 2012; Morrissey & Ferguson, 2011; Roy, Roy, Grant, & Bergeron, 2013; Sneddon, 2003; Steingrimsson & Grant, 2003; Wilson & Godin, 2009; Wright, Nakamichi, Krause, & Butlin, 2006). In general, studies of mobile animals usually find that some individuals move longer distances than the majority of individuals and that variability in movement is the direct result of heritable differences in movement rate, which in turn has fitness consequences (Fraser, Gilliam, Daley, Le, & Skalski, 2001).

In this study, our objective was to determine the extent to which the evolution of movement rate improves the efficacy of large marine reserves in protecting pelagic fish populations. Our hypothesis was that heritable variation among individuals in mean movement distance, combined with higher mortality among individuals that move outside of marine reserves (i.e., natural selection), should promote the evolution of low movement rates, and thereby result in more individuals staying within the boundaries of marine reserves. We simulated individual‐level processes (births, deaths, and movement) in a spatially explicit (i.e., grid‐based) model combining population dynamics with evolutionary dynamics. We chose four distinct parameter sets, informed by empirical data, to represent the range of life histories (e.g., short life span and small size vs. longer life span and large size) among tuna species (skipjack tuna vs. Atlantic bluefin tuna) and shark species (great white sharks vs. spiny dogfish). We predicted that the evolution of decreased movement rate following the establishment of marine reserves would lead to higher within‐reserve population size relative to situations with no heritable variation in movement rate. We also predicted that higher fisheries‐induced mortality (e.g., overfishing) would lead to faster evolution of reduced movement.

## 2 Methods

**Figure 1:**
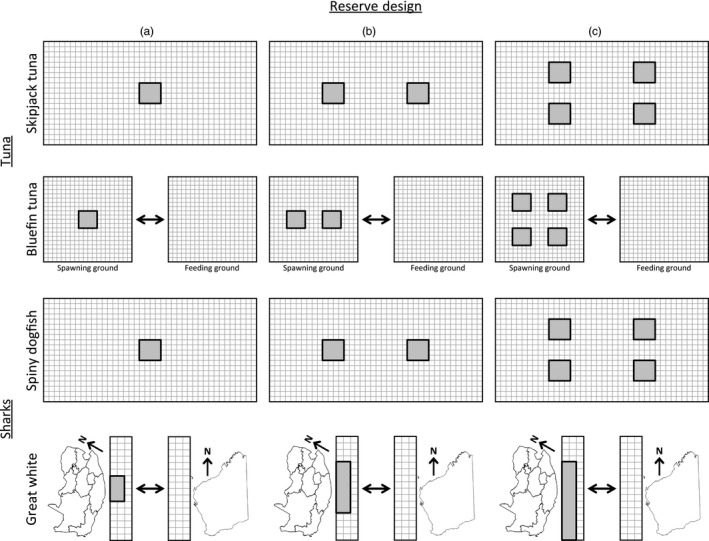
Simulation grids and reserve designs (a–c) used for simulations involving tuna and shark species. Arrows indicate that migration occurs between feeding and spawning grounds for bluefin tuna or, for great white sharks, between a local population (South Africa—on the left of each panel) and a nonlocal population (Western Australia—on the right). Dark gray areas indicate reserve locations. Each grid cell represents a 100 × 100 NM patch of ocean

Our model builds upon the individual‐based model of marine reserves developed by Miethe et al. (2009), which considered the evolution of movement rate between two patches and the evolution of size at maturity for a haploid, asexual organism. We also considered the evolution of movement rate, but we did so in a spatially explicit framework. Furthermore, we modeled fish more realistically as diploid, sexually reproducing organisms. We tracked individual‐level processes across multiple patches arranged in a grid, wherein each patch (or grid cell) is analogous to a square area of ocean of 100 NM in length (Figure 1). The size and arrangement of grids were adjusted based on species‐specific data. For example, because the “tuna belt” in the Pacific Ocean consists of about 8,000,000 NM^2^ stretching about 2,000 NM from latitude 20**°**N to 20**°**S and about 4,000 NM from 150**°**E to 140**°**W, we used a 20 × 40 grid to represent the Pacific Ocean tuna belt. Typically, a single marine reserve encompassing the entire Exclusive Economic Zone (EEZ) of an oceanic island consists (roughly) of a circle of 400 NM in diameter, which we modeled as a square reserve of equal length (16 cells in a 4 × 4 configuration).

We tracked the dynamics at a single two‐allele gene causing variation among fish in their movement distances. Each individual's movement was determined by its diploid genotype: *AA*,* Aa*, or *aa*, with a 10% initial frequency of allele *a* (the low‐movement allele). Note that these genotypes did not cause a switch between migratory and nonmigratory behavior, but rather affected the net distance travelled, which might be due to changes in angles of movement, rather than total distance traveled. Hence, we do not consider that life history changes necessarily accompany these shifts in net distance traveled, although further work could examine pleiotropic effects or subsequent evolution of life history alongside movement rate evolution. Each genotype conferred a mean (or expected) net movement distance, μ_*i*_ (Table 1), with the actual net movement distance of an individual in NM drawn randomly from a negative binomial distribution with variance equal to μ_*i*_ + μ_*i*_^2^/2. The resulting distribution of movement distances (based on the negative binomial) is similar to a gamma distribution with rounding, which is appropriate for our purposes because (1) the range is appropriate (including fish that remain in the same cell and fish that can move a large distance), and (2) the distribution closely resembles empirically observed distributions of movement distances (Sibert & Hampton, 2003). Additive gene action was assumed (dominance coefficient: dc = 1/2). Movement direction, θ, was determined by a random draw from a uniform distribution ranging from 0 to 2π (0 representing east and π/2 representing south). We then calculated the net distance moved in the longitudinal direction (cosθ × distance) and latitudinal direction (sinθ × distance). If the distance in either direction brought an individual to the edge of the grid, any additional distance was reflected in the opposite direction. Each individual had a nonzero probability of remaining within the same cell (given by the probability of drawing a net movement distance smaller than the distance from the center of a cell to its edge), and otherwise it moved to other cells probabilistically, according to the direction and angle of movement. Modeling movement probabilistically across a grid greatly increased the speed of the simulations; given the relatively large number of grid cells, the loss of accuracy is not expected to be substantial, relative to explicitly tracking movement of each individual in continuous space (as noted in Berec, 2002; Mooij & DeAngelis, 2003).

**Table 1:** List of parameter values used in simulations. See main text and footnotes for justification and sources of parameter values

Parameter (units)	Value(s)				Description
	Skipjack	Bluefin	Dogfish	G.w. shark	
*M* (year^−1^)	1	0.16	0.16	0.11	Natural mortality rate for adults^a^
*s* (year^−1^)	0.37	0.85	0.85	0.9	Natural survival proportion for adults (=e^‐*M*^)
*s*_*b*_ (year^−1^)	0.37	0.37	0.85	0.85	Natural survival proportion for fry or pup
*dd*	0.0005	0.0005	0.005	0.005	Factor determining density dependence of fry or pup survival^b^
*maturity.age* (years)	1.5	8	8	10	The average age at which individuals mature^c^
*f* (year^−1^)	1500	1500	5	5	Fecundity (mean number of offspring per adult female)
*fished* (year^−1^)	0.32, 0.8	0.075, 0.75	0.075, 0.225	0.05, 0.2	Fishing mortality proportion
*mig*	n.a.	2, 6	n.a.	2	Mean number of years spent in feeding grounds before returning to spawning grounds
μ_*AA*_ (NM·year^−1^)	600, 400, 200.	800, 600, 400	200, 100	800, 600	Mean distance swum by individuals with *AA* genotype
μ_*aa*_ (NM·year^−1^)	600, 400, 200, 100,	800, 600, 400, 200	200, 100, 50, 25	800, 600, 400, 200	Mean distance swum by individuals with *aa* genotype
dc	0.5	0.5	0.5	0.5	Dominance coefficient: individuals of genotype *Aa* move an average distance of μ_*Aa*_ = μ_*aa*_ + dc × (μ_*AA*_ * *− μ_*aa*_).
*init.a*	0.1	0.1	0.1	0.1	Initial frequency of allele *a* in all grid cells

a The mortality value for each species was selected from within the range of values reported on fishbase.org.

b The effect of density on survival (i.e., the value of the *dd* parameter) was initially set to the value used in Miethe et al. (2009) and then adjusted (for the shark species), after running several pilot simulations, to a value that resulted in population persistence without any fishing pressure.

c See Table 2 for the source of these age of maturity values.

Sex (male or female) was assigned randomly to each individual at birth. At the time of reproduction, mating occurred only if there was at least one mature male within the grid cell of a female, with all fertilized females producing the same mean number of eggs (determined by the *fecundity* parameter in Table 1). Within a cell, the number of eggs carrying the *A* allele was drawn randomly from a Poisson distribution with a mean of *fecundity* times the number of *AA* females + *fecundity* times the number of *Aa* females/2, and the number of eggs carrying the *a* allele was similarly based on the number of *aa* and *Aa* females. We assumed no sperm limitation within cells containing a male, and the allele frequency among sperm was determined by the allele frequency among males in the cell. Allowing more individual variance in fecundity (e.g., by using a negative binomial distribution) would increase genetic drift (i.e., decrease effective population size) but should not have any other effect on our results. The number of zygotes (and, subsequently, hatchlings and fry) of each genotype was determined by randomized binomial trials, given the number of eggs carrying each allele and the allele frequency among sperm. The low fecundity values used in our simulations (Table 1) relative to the actual number of eggs produced by tuna (e.g., a large bluefin tuna female can produce hundreds of millions of eggs in a single spawning bout) were meant to reflect the high mortality between the egg and fry stage. Ovoviviparous shark species, on the other hand, typically produce few offspring, and shark pups likely have a higher survival rate than tuna fry (Compagno, 1984, 2002).

There were three age classes in our model: fry, juveniles, and adults. Fry either died or survived and were then recruited to the juvenile age class in the year of their birth (i.e., all fry were age 0). We assumed no larval dispersal, meaning that fry did not move outside of the cell in which they were spawned. Fry survival was negatively density dependent based on the number of fry in the grid cell:

$$\text{density}\text{‐}\;\;\text{dependent}\;\text{survival}=s_{b}/\left(1+dd\times \text{number}\;\text{of}\;\text{fry}\right),$$

where *s*_*b*_ is the proportion of fry that would survive if there were no density dependence, and *dd* determines the magnitude of density dependence (Table 1). We then used randomized binomial trials to determine fry survival within the cell, given the number of fry in the grid cell and the probability of density‐dependent survival. To determine juvenile and adult survival, we used binomial trials, where the probability of survival per year was *s* (Table 1), and the number of trials was equal to the number of juveniles or mature adults in the grid cell. Of the surviving juveniles in the grid cell, the number that became reproductively mature (entering the adult age class) was again drawn from a binomial, with a probability of maturation, *p*, based on the *maturity.age* parameter (Table 1): *p* = 1/*maturity.age*.

In our model, the rate of fishing mortality (i.e., the proportion harvested) was highest in the cells with the highest fish abundance. Grid cells containing an average number of individuals (*mean.patch.pop*) experienced a rate of fishing mortality of *fished* (Table 1). The *fished* parameter determined fishing mortality as a proportion of the population remaining after natural mortality. A value of *fished* = ½(1−*s*), representative of relatively low fishing pressure, was used for all species. Results were then contrasted against a high fishing pressure, where *fished* was determined from pilot simulations to be the maximum value that allowed persistence for at least 50 years; these were determined to be *fished* = 0.8 for skipjack‐like life histories, 0.75 for bluefin tuna, 0.225 for dogfish, and 0.2 for great white sharks. We chose a hyperbolic function to describe the actual fishing pressure, *f*_*i,j*_, experienced in each cell depending on the fish abundance in that cell, *pop*_*i,j*_

$$f_{i,j}=pop_{i,j}/\left[mean.patch.pop\times \left(1/fished-1\right)+pop_{i,j}\right].$$

This function was chosen for the following properties. If every population had the same mean population size, then the fishing pressure in each is *fished*. For populations much smaller than the mean, however, the fishing pressure is assumed to rise linearly with the local population size (i.e., *f*_*i,j*_ ~ *constant* times *pop*_*i,j*_, where *constant* = 1/(*mean.patch.pop*(1/*fished *− 1)) when *pop*_*i,j*_ << *mean.patch.pop*). Finally, this function ensured that the maximum fishing pressure never exceeded 100%, even in populations of very large size (*pop*_*i,j*_ >> *mean.patch.pop*). Fishing mortality in each grid cell was drawn randomly from a binomial distribution, where the probability of being caught was *f*_*i,j*_, and the number of trials was equal to the number of individuals in the grid cell after natural mortality. Fishing pressure was assumed to be sufficiently high that adults are effectively released from density‐dependent competition, and, aside from fry survival, we did not include density‐dependent processes in our model. A direction for future work might be to relax this assumption (e.g.,Gårdmark, Jonzén, & Mangel, 2006), especially in cases of highly successful MPAs where local densities can rebound and shift the life history stage at which density dependence is strongest.

After reserve establishment, the grid cells that were designated as reserves experienced no fishing mortality (“no‐catch” reserves). Total fisheries pressure was, however, kept constant (i.e., fishing effort was displaced) by considering only nonreserve patches in the calculation of *mean.patch.pop*, and by increasing mean fishing mortality outside of the reserves in proportion to the area of the reserves (i.e., if a fraction *x* of cells were designated as reserves, the adjusted mean fishing mortality in the remaining cells was set to *fished*/(1 − *x*). Fishing mortality continued to track fish abundance after reserve establishment. Consequently, any spillover of fish from reserves to neighboring cells led to higher abundance and hence higher fishing mortality adjacent to reserves.

The simulations proceeded in yearly time increments with the following order of events: spawning–natural mortality–recruitment–fishing–movement. The initial frequency of the *a* allele (*init.a*) was set to 0.1 in all grid cells, and all grid cells started with the same initial population size (1,000 for skipjack and dogfish simulations, 500 for bluefin and great white shark simulations). The heritability at the beginning of each simulation (*h* ^2^ = *V*_A_/*V*_P_) was estimated by randomly drawing movement distances in proportion to the genotype frequencies to obtain an estimate of total phenotypic variation (*V*_P_) and using the one‐locus model (Falconer & MacKay, 1996) to calculate additive genetic variance (*V*_A_): *V*_A_ = 2(1 − *q*)*q*α^2^, where *q* is the frequency of allele *a* (i.e., *init.a*, Table 1), and α is the average allelic effect: α = (μ_*AA*_ − μ_*aa*_)/2 for the additive case considered here (dc = 1/2). We note that alternative genetic models, such as quantitative genetic models that assume constant heritability, may be less appropriate, especially for evolution in small populations, where evolutionary responses might be strongly constrained/limited to the dynamics of a few loci. Population size was allowed to stabilize across all cells for 50 years prior to the commencement of fishing. We then simulated 50 years of fishing prior to the establishment of reserves. After reserve establishment at year 100, we ran the simulations for 50 years (except where noted), which is a time frame of relevance for resource managers (at least, more so than longer evolutionary time frames). The efficacy of reserves was evaluated by comparing mean within‐reserve population density (i.e., individuals per cell) at year 150 (i.e., 50 years postestablishment) to population density at year 150 in control simulations without any reserves. We ran ten replicates of each parameter combination. For each species (skipjack tuna, bluefin tuna, dogfish, and great white shark), for both levels of fishing mortality (low or high), we evaluated the effect of reserve number (or reserve size in the case of great white sharks) and movement distance on within‐reserve population density and frequency of allele *a* 50 years after reserve establishment. We used Dunnett's test of multiple comparisons (Dunnett, 1955) to compare every parameter combination (i.e., each unique combination of reserve number or size, μ_*AA*_ and μ_*aa*_) to the control case (with no reserves). All simulations and analyses were performed using the R programming language (Hothorn, Bretz, & Westfall, 2008; R Core Team 2014). The R code used to run the simulations is provided in Supporting Information.

### 2.1 Model parameters

We chose parameter sets (Table 1) that would reflect the range of life histories among tuna species and shark species. We began by looking at von Bertalanffy life history parameters (von Bertalanffy, 1938), and we chose species with extreme values of growth in length (*k*) and asymptotic body length (*L*_*∞*_) relative to the ranges of these values for tunas and sharks (Table 2). We based our species selection on median parameter values reported for individual species in FishBase (Froese & Pauly, 2016). We selected skipjack tuna, *Katsuwonus pelamis*, to represent tuna species with relatively short lifespan (high *k*) and small size (low *L*_*∞*_), and Atlantic bluefin tuna, *Thunnus thynnus*, to represent tuna species with relatively longer lifespan (low *k*) and large size. All shark species are slow growers, and we selected spiny dogfish, *Squalus acanthias*, to represent a small shark species (Orlov, Kulish, Mukhametov, & Shubin, 2011), and great white sharks, *Carcharodon carcharias*, to represent a large species (Wintner & Cliff, 1999). We drew species‐specific parameter estimates for our models from FishBase.org and other literature sources, as described below (see also Tables 1 and 2). Given the lack of information in the literature that would inform values of the fry or pup survival parameter (*s*_*b*_), we based the value for this parameter on two *a priori* assumptions. First, we assumed a single value for both tuna species and a single value for both shark species (because of the lack of any known substantive life history differences among species at this early life stage). Second, we assumed that fry or pup survival was not higher than adult survival. We then ran several pilot simulations and chose values for the *s*_*b*_ parameter that resulted in population persistence and stability in the absence of any fishing pressure.

**Table 2:** Range of selected life history characteristics (age at maturity, asymptotic length, and growth rate) among tuna and shark species (from fishbase.org; Compagno, 1984; Fromentin & Fonteneau, 2001; Compagno, 2002)

Species	*T*_*M50%*_ (years)	*L*_*∞*_ (cm)	*k* (year^−1^)
Tunas			
Skipjack tuna	1.5	83	0.8
Atlantic little tuna	1.5	115	0.2
Yellowfin tuna	2.8	186	0.8
Bigeye tuna	3.5	203	0.2
Atlantic sailfish	3	236	1.1
Atlantic white marlin	3	261	0.6
Albacore (North Atlantic)	4.5	134	0.2
Swordfish (North Atlantic)	5	252	0.1
Bluefin tuna (Northeast Atlantic and Mediterranean)	4.5	330	0.1
Southern bluefin tuna	8	220	0.2
Sharks			
Picked dogfish	8	116	0.1
Great white shark	10	653	0.1

### 2.2 Skipjack tuna

The majority of skipjack tuna catch worldwide occurs in the “tuna belt”, which is an area extending approximately 2,000 NM from Wake Island in the north to New Caledonia in the south, and 4,000 NM from Indonesia in the west to the Marquesas in the east. We, therefore, used a 20 × 40 grid for our simulations involving skipjack tuna (Figure 1). Sibert and Hampton (2003) calculated lifetime displacement (distance from the point of release to the point of capture of a tagged fish during its lifetime) of adult skipjack tuna, based on tagging data from the South Pacific, using a spatially explicit advection–diffusion reaction model (Sibert, Hampton, Fournier, & Bills, 1999). Given a median net lifetime displacement for skipjack tuna of between 411 and 471 NM (Sibert & Hampton, 2003), we estimated an annual displacement (for a species with a 2–4 year life span) between 110–235 NM. In any case, median yearly displacement is likely less than the lifetime median values. We selected values for μ_*AA*_ (i.e., the expected yearly movement distance for an individual homozygous for allele *A*) of 600, 400, and 200 NM and values of μ_*aa*_ (always less than or equal to μ_*AA*_) of 600, 400, 200, and 100 NM (Table 1). We contend that this range of values likely brackets the real annual displacement distance for skipjack tuna, and we point out that a negative binomial distribution with a mean value of 600, 400, or 200 NM and a variance of μ + μ^2^/2 is qualitatively very similar to the observed distribution of tag displacements reported in Sibert and Hampton (2003). Heritability at the beginning of these simulations varied from approximately 0.01 to 0.06, except when μ_*AA*_ = μ_*aa*_ (no heritability). For skipjack tuna, we compared the effects of establishing one, two, or four reserves each of size 400 × 400 NM (Figure 1).

### 2.3 Bluefin tuna

Bluefin tuna and other tuna in the genus *Thunnus*, such as bigeye tuna (*T. obesus*) and albacore (*T. alalunga*), are highly migratory species (FAO 1994). Atlantic bluefin tuna (*T. thynnus*), for example, migrate to spawn in the Mediterranean Sea or Gulf of Mexico in the spring or summer, but immature individuals and nonspawning adults spend the majority of their time in productive waters off the east coast of North America (e.g., North Carolina's Outer Banks and Flemish Cap) and, to a lesser extent, in the mid‐Atlantic (Block et al., 2005). Pacific bluefin tuna, *T. orientalis*, have the largest individual ranges of any *Thunnus* species, migrating from spawning grounds around Japan and the Philippines to productive waters off the west coast of North America (Bayliff, 1994). Although total ranges for these species (including transoceanic migrations) are very large, individuals may spend substantial periods of time in much more restricted areas off the coasts of North America or in the western Pacific (Bayliff, 1994; Boustany, Matteson, Castleton, Farwell, & Block, 2010). Therefore, in our simulations for bluefin tuna, we modeled migration and movement as two separate processes. All individuals migrated from the spawning ground to the feeding ground and back prior to maturity (Figure 1). The spawning and feeding grounds were simulated as separate 20 × 20 grids linked by migration (Figure 1). The rate of movement was allowed to evolve within each area, but not the rate of migration between spawning and feeding grounds, which was determined by the *mig* and *maturity.age* parameters (Table 1). After birth, individuals migrated from the spawning to the feeding ground at (on average) *maturity.age*–*mig*, then migrated back to the spawning ground after (on average) *mig* years in the feeding grounds. The average time spent in the feeding and spawning grounds (and, hence, the value of the *mig* parameter) was drawn from Bayliff (1994). Migration between spawning and feeding grounds occurred in each time step prior to spawning (such that the yearly series of events was: migration–spawning–natural mortality–recruitment–fishing–movement). The earliest an individual could have spawned (if they migrated to the feeding ground in year 0 and returned to the spawning ground in year 1) was at age 2. Adults left the spawning ground in the time step immediately after spawning and then spent (on average) *mig* years in the feeding grounds before returning once again to the spawning grounds. Movement was modeled as a process that only occurred within the spawning or feeding grounds. We selected values for μ_*AA*_ of 800, 600, and 400 NM and values of μ_*aa*_ of 800, 600, 400, and 200 NM (Table 1). We contend that this range of values likely brackets the real annual displacement distances for bluefin tuna within either their feeding or spawning grounds (Bayliff, 1994; Block et al., 2005; Boustany et al., 2010). Heritability at the beginning of these simulations varied from approximately 0.006 to 0.06, except when μ_*AA*_ = μ_*aa*_ (no heritability). For bluefin tuna, we compared the effects of establishing one, two, or four reserves, each of size 400 × 400 NM, within the spawning grounds (Figure 1).

### 2.4 Spiny dogfish

Given the implications of climate change models for marine biodiversity (e.g., Cheung et al., 2009), the distribution of spiny dogfish over the course of the next half century is projected to include continental shelves, seamounts, and islands throughout the Pacific, including the coasts of Australia and New Zealand, as well as the Solomon Islands and the islands of French Polynesia (Aquamaps 2013). We, therefore, used a 20 × 40 grid to simulate an area across the South Pacific, between southeast Australia, New Zealand, French Polynesia, and the Solomon Islands. Tagging data suggest that spiny dogfish tend to move very little (Carlson, Hoffmayer, Tribuzio, & Sulikowski, 2014; McFarlane & King, 2003). We selected values for μ_*AA*_ of 200 and 100 NM and values of μ_*aa*_ of 200, 100, 50, and 25 NM (Table 1). Again, this range of values likely brackets the real annual displacement distance for spiny dogfish, barring occasional and irregular long distance migrations (Carlson et al., 2014; McFarlane & King, 2003). Heritability at the beginning of these simulations varied from approximately 0.03 to 0.07, except when μ_*AA*_ = μ_*aa*_ (no heritability). For spiny dogfish, we compared the effects of establishing one, two, or four reserves, each of size 400 × 400 NM (Figure 1).

### 2.5 Great white sharks

Great white sharks are typically a coastal species, with major centers of abundance off the coastal waters of Baja California, Australia–New Zealand, and South Africa (Bonfil et al., 2005; Compagno, 2002). White sharks are not considered a highly migratory species (FAO 1994), but they are known to make regular transoceanic migrations, perhaps for the purposes of mating (Bonfil et al., 2005). Indeed, white sharks have been observed to make the 20,000 km round trip between South Africa and Australia in less than nine months (Bonfil et al., 2005). Great white sharks also move extensively within their home range. For example, a tagging study revealed that approximately 25% of individuals tagged off the Western Cape of South Africa travelled 2,000 km to KwaZulu‐Natal (near the border of Mozambique), and 12.5% made the return trip within a single year (Bonfil et al., 2005). In our simulations for great white sharks, we used a 16 × 3 grid to represent the coastline off South Africa and Mozambique, extending 300 NM from shore (Figure 1). We biased movement within this coastal region to be along the coast (as opposed to seaward) by converting θ to the root of the function x−cosx×sinx−θ within the interval 0 < *x *< 2π (leading to >80% of movements along the coast). We selected values for μ_*AA*_ of 800 and 600 NM and values of μ_*aa*_ of 800, 600, 400, and 200 NM (Table 1), chosen to bracket the likely annual displacement distances for great white sharks within their natal population (Bonfil et al., 2005). Heritability at the beginning of these simulations varied from approximately 0.006 to 0.06, except when μ_*AA*_ = μ_*aa*_ (no heritability). Note that, using a negative binomial distribution for movement with a mean of 800 or 600 NM and a variance of μ + μ^2^/2, approximately 25% of individuals would travel at least 1,000 NM, which is on the order of observed movement distances along the coast of South Africa (Bonfil et al., 2005). We also allowed long distance (transoceanic) migration to another coastal population (e.g., off the Australian coast). All individuals spent an average of two years in their natal population (i.e., South Africa) before migrating, and migrants always returned to their natal population within a year. For great white sharks, we compared the effects of protecting 8.8%, 16.6%, or 25% of the total grid, placed solely within South Africa's EEZ, corresponding to a reserve that spans 400, 800, or 1,200 NM of coastline, respectively (Figure 1).

## 3 Results

### 3.1 Skipjack tuna

**Figure 2:**
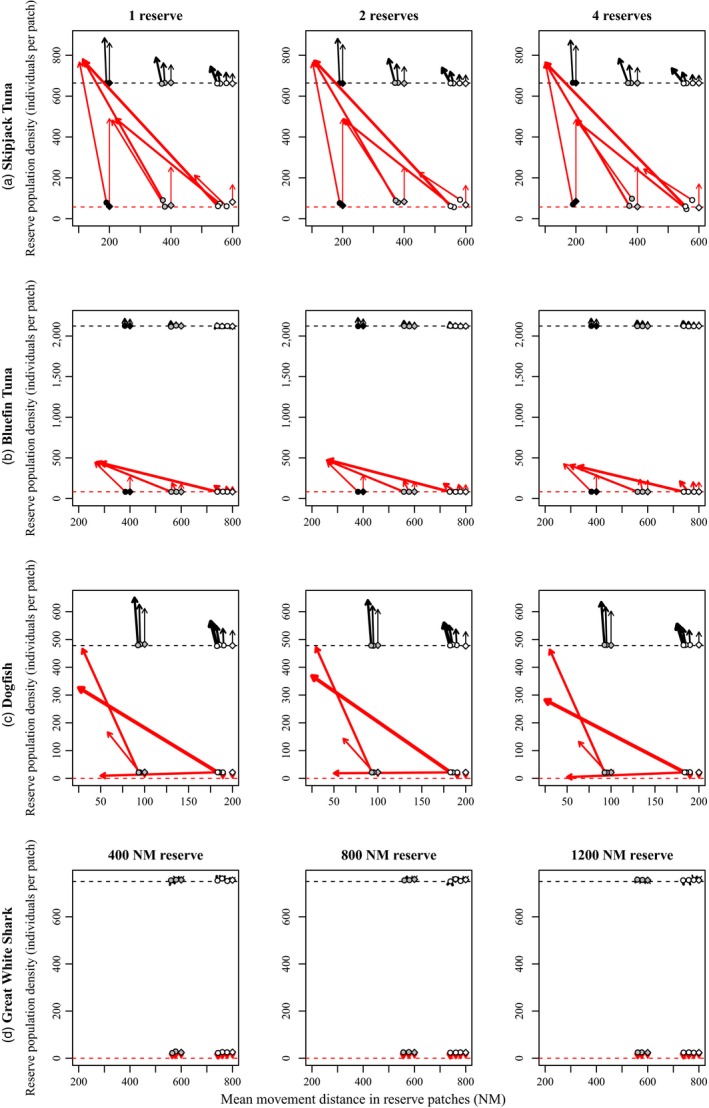
Evolution of movement rate and changes in within‐reserve population density for skipjack tuna (a), bluefin tuna (b), dogfish (c), and great white shark (d). Each arrow corresponds to one parameter combination and shows the evolution of movement rate (horizontal axis) and the associated change in population density (vertical axis) 50 years after reserve establishment (at the arrow head) relative to prereserve conditions (circle or diamond symbols at the tail of the arrow). Diamond symbols indicate prereserve conditions for scenarios with no heritable variation in movement distance (and hence no possibility of evolution). Symbol color indicates the value of the μ_*AA*_ parameter for each parameter combination (skipjack tuna: white = 600 NM, gray = 400 NM, black = 200 NM, bluefin tuna: white = 800 NM, gray = 600 NM, black = 400 NM; dogfish: white = 200 NM, gray = 100 NM; great white shark: white = 800 NM, gray = 600 NM). Values of the μ_*aa*_ parameter (always ≤μ_*AA*_) were 600, 400, 200, or 100 NM for skipjack tuna, 800, 600, 400, or 200 NM for bluefin tuna, 200, 100, 50, or 25 NM for dogfish, and 800, 600, 400, or 200 NM for great white shark. Arrow line weight increases with increasing heritable variation in movement rate (i.e., thickest when μ_*aa*_ **<< μ_*AA*_, thinnest when μ_*aa*_ = μ_*AA*_), and line color indicates fishing mortality (black = low; red = high). Dashed lines show the equilibrium population sizes without reserves. All data are averaged over 10 replicate simulations

**Figure 3:**
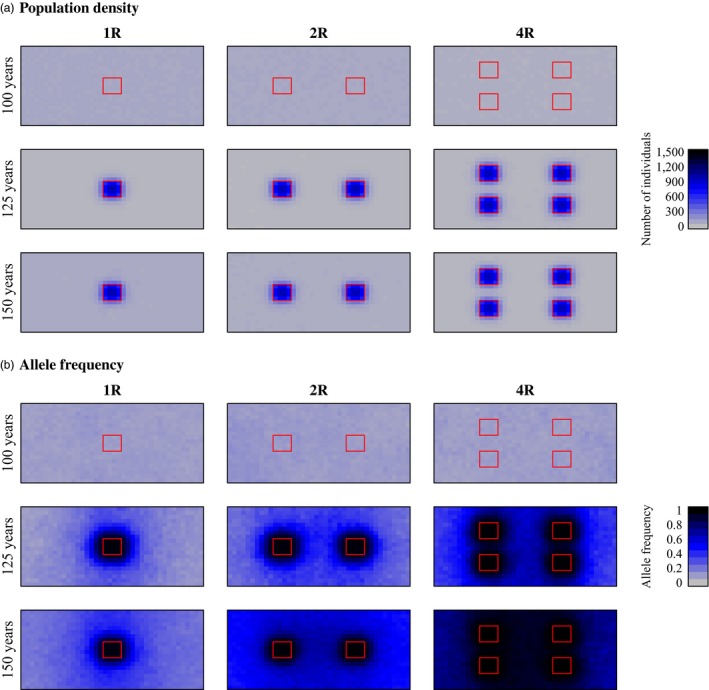
Examples of the change in population density (a: individuals per patch) and evolution of movement rate (b: frequency of the low‐movement‐rate allele) for skipjack tuna. Reserves were established in year 100. Red boxes indicate locations of reserves for cases with one reserve (1R, left column), two reserves (2R, middle column), or four reserves (4R, right column). Other parameter values in these simulations are as follows: μ_*AA*_ = 400 NM; μ_*aa*_ = 100 NM;* fished* = 0.8. Data are averaged over 10 replicate simulations

Within‐reserve population densities of skipjack tuna were significantly higher than population densities without reserves (Table 3, Figure 2a, Figs S1 and S2). In the scenarios with high fishing mortality, the effect of marine reserves was much more substantial than in equivalent scenarios with low fishing pressure (Figure 2a, Figs S1 and S2). Regardless of fishing mortality, all combinations of reserve number and movement distances resulted in significant increases in within‐reserve population density relative to the control case without reserves (Table 3). Regardless of fishing mortality or number of reserves, marine reserves were more effective when movement distances (μ) were lowest (Figure 2a, Figs S2 and v). Within‐reserve population densities of skipjack tuna were higher than population densities outside reserves, and marine reserves also lead to increases in population density in nonreserve grid cells adjacent to marine reserves (Figure 3a).

**Table 3:** Population size and frequency of low‐movement allele for skipjack tuna 50 years after establishment of marine reserves (average over 10 replicate simulations). Values that are significantly higher than control values (with no reserves), according to Dunnett's test of multiple comparisons to a control, are shown in bold and italic type (*p *< .05). Allele *A* was assumed to act additively throughout (columns in gray indicate cases with no heritability in migration rate)

Fishing mortality	Number of reserves	Movement phenotypes (mean movement distance in NM for the genotypes μ_*AA*_; μ_*aa*_)								
		600; 600	600; 400	600; 200	600; 100	400; 400	400; 200	400; 100	200; 200	200; 100
		Within‐reserve population density (individuals per patch)								
Low	(control) 0	664 (*SD *= 4.88)								
	1	***710***	***716***	***722***	***743***	***753***	***762***	***789***	***861***	***885***
	2	***703***	***705***	***719***	***738***	***744***	***757***	***782***	***852***	***881***
	4	***702***	***704***	***716***	***735***	***740***	***751***	***783***	***847***	***873***
High	(control) 0	57 (*SD *= 24.00)								
	1	***166***	***212***	***490***	***770***	***252***	***482***	***781***	***490***	***766***
	2	***163***	***226***	***484***	***773***	***252***	***489***	***772***	***488***	***763***
	4	***170***	***242***	***481***	***764***	***253***	***485***	***762***	***486***	***763***
		Within‐reserve frequency of allele *a*								
Low	(control) 0	0.10 (*SD *= 0.005)								
	1	0.10	0.10	***0.12***	***0.14***	0.10	***0.12***	***0.16***	0.10	***0.16***
	2	0.10	***0.11***	***0.13***	***0.15***	0.10	***0.12***	***0.17***	0.10	***0.17***
	4	0.10	***0.11***	***0.13***	***0.18***	0.10	***0.13***	***0.20***	0.10	***0.18***
High	(control) 0	0.10 (*SD *= 0.024)								
	1	0.09	***0.62***	***0.95***	***0.97***	0.11	***0.96***	***0.96***	0.09	***0.97***
	2	0.12	***0.76***	***0.99***	***0.98***	0.11	***0.98***	***0.98***	0.10	***0.98***
	4	0.10	***0.89***	***1.00***	***1.00***	0.11	***1.00***	***1.00***	0.11	***0.99***

Consistent with our predictions, decreased movement rate evolved following the establishment of marine reserves (Figures 2a and 3b, Figs S3 and S4). There were significant increases in the frequency of allele *a* (the low‐movement‐rate allele) in all cases except when there was no heritable variation (i.e., when μ_*AA*_ = μ_*aa*_) or with the lowest nonzero heritability and the lowest selection pressure (i.e., when fishing mortality was low, there was one reserve, μ_*AA*_ = 600 NM, and μ_*aa*_ = 400 NM; Table 3). Movement evolution was much more extensive with high fishing mortality than with low fishing mortality (Figure 2a, Figs S3 and S4). Greater genetic changes were seen with higher heritable variation (i.e., when μ_*AA*_ − μ_*aa*_ was large; Figure 2, Figs S3 and S4). These evolutionary reductions in movement rate (i.e., increases in the frequency of allele *a*) were associated with substantial increases in within‐reserve population sizes over the course of the 50 years following reserve establishment (Figure 2a). The spatial pattern of allele frequency change indicates that the evolution of decreased movement was concentrated in and around reserves (Figure 3b).

### 3.2 Bluefin tuna

**Figure 4:**
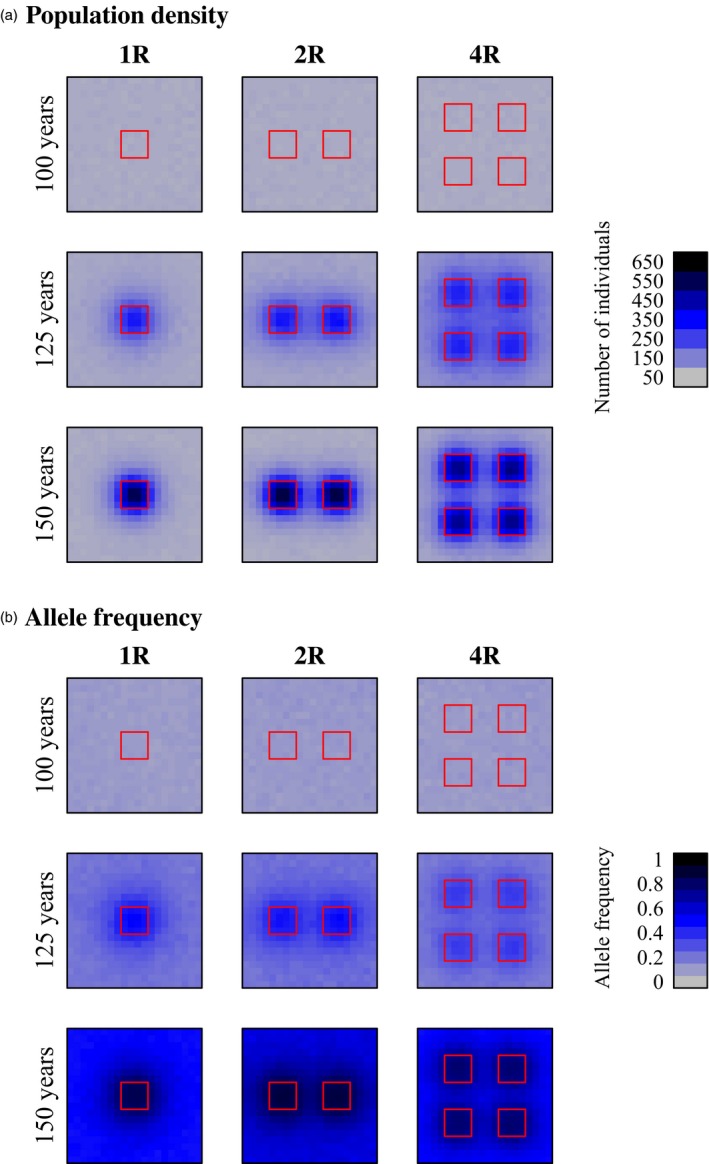
Examples of the change in population density (a: individuals per patch) and evolution of movement rate (b: frequency of the low‐movement‐rate allele) for bluefin tuna across the spawning grounds (feeding grounds not shown). Reserves were established in year 100. Layout and details as in Figure 3. Other parameter values in these simulations are as follows: μ_*AA*_ = 600 NM; μ_*aa*_ = 200 NM;* fished* = 0.75

Within‐reserve population densities of bluefin tuna were significantly higher than population densities without reserves (Table 4, Figure 2b, Figs S5 and S6). In scenarios with high fishing mortality, marine reserve had a more substantial effect on population density than in equivalent scenarios with low fishing mortality (Figure 2b, Figs S5 and S6). Regardless of fishing mortality, all combinations of reserve number and movement distances resulted in significant increases in within‐reserve population density relative to the control case without reserves (Table 4). Regardless of fishing mortality or number of reserves, marine reserves were most effective when movement distances were lowest (Figure 2b, Figs S5 and S6). Within‐reserve population densities of bluefin tuna were higher than population densities outside reserves, and marine reserves also lead to increases in population density in nonreserve grid cells adjacent to marine reserves (Figure 4a).

**Table 4:** Population size and frequency of low‐movement allele for bluefin tuna 50 years after establishment of marine reserves (average over 10 replicate simulations). Values that are significantly higher than control values (with no reserves), according to Dunnett's test of multiple comparisons to a control, are shown in bold and italic type (*p *< .05). Allele *A* was assumed to act additively throughout (columns in gray indicate cases with no heritability in migration rate)

Fishing mortality	Number of reserves	Movement phenotypes (mean movement distance in NM for the genotypes μ_*AA*_; μ_*aa*_)								
		800; 800	800; 600	800; 400	800; 200	600; 600	600; 400	600; 200	400; 400	400; 200
		Within‐reserve population density (individuals per patch)								
Low	(control) 0	2013 (*SD *= 8.77)								
	1	***2140***	***2150***	***2151***	***2152***	***2166***	***2170***	***2181***	***2209***	***2215***
	2	***2152***	***2158***	***2156***	***2166***	***2171***	***2176***	***2186***	***2211***	***2224***
	4	***2173***	***2169***	***2173***	***2179***	***2177***	***2185***	***2189***	***2209***	***2216***
High	(control) 0	83 (*SD *= 13.42)								
	1	***146***	***149***	***156***	***438***	***181***	***191***	***451***	***270***	***445***
	2	***169***	***171***	***182***	***475***	***203***	***214***	***472***	***284***	***450***
	4	***202***	***204***	***212***	***393***	***226***	***234***	***410***	***295***	***425***
		Within‐reserve frequency of allele *a*								
Low	(control) 0	0.100 (*SD *= 0.0014)								
	1	0.100	0.100	0.101	***0.102***	0.099	0.101	***0.102***	0.100	***0.102***
	2	0.099	0.100	0.100	***0.103***	0.101	0.100	***0.103***	0.100	***0.102***
	4	0.100	0.100	0.101	***0.102***	0.099	0.101	***0.102***	0.100	***0.103***
High	(control) 0	0.100 (*SD *= 0.0310)								
	1	0.096	0.118	***0.176***	***0.853***	0.096	***0.180***	***0.822***	0.099	***0.680***
	2	0.091	***0.139***	***0.213***	***0.893***	0.098	***0.208***	***0.854***	0.106	***0.698***
	4	0.094	0.112	***0.185***	***0.782***	0.105	***0.148***	***0.754***	0.103	***0.633***

We observed less evolution of movement rate over 50 years in bluefin tuna simulations than in skipjack tuna simulations, even at similarly high fishing mortalities. With low fishing mortality (*fished* = 0.075), very little evolution of movement rate was observed (Figure 2b, Fig. S7), and there were significant increases in the frequency of allele *a* only when heritability was highest (i.e., when μ_*aa*_ = 200 NM; Table 4). Consistent with our prediction, however, there was substantial evolution of movement rate when fishing pressure was high (*fished* = 0.75; Figure 2b, Fig. S8), and greater evolutionary changes were seen with higher heritable variation (i.e., when μ_*AA*_ − μ_*aa*_ was large; Table 4; Figure 2b, Fig. S8). There were significant increases in the frequency of allele *a* (the low‐movement‐rate allele) in all cases except when there was no heritable variation (i.e., when μ_*AA*_ = μ_*aa*_) or very low heritable variation (i.e., when μ_*AA*_ = 800 NM, and μ_*aa*_ = 600 NM; Table 4). The spatial pattern of allele frequency change indicates that the evolution of decreased movement was concentrated in and around reserves (Figure 4b). Varying the length of time spent in the feeding grounds prior to returning to the spawning grounds (*mig* = 2 or 6) did not have a qualitative effect on the patterns described above, except that a longer residency in the feeding grounds (*mig* = 6) reduced the efficacy of marine reserves, which were situated in the spawning grounds.

### 3.3 Dogfish

**Figure 5:**
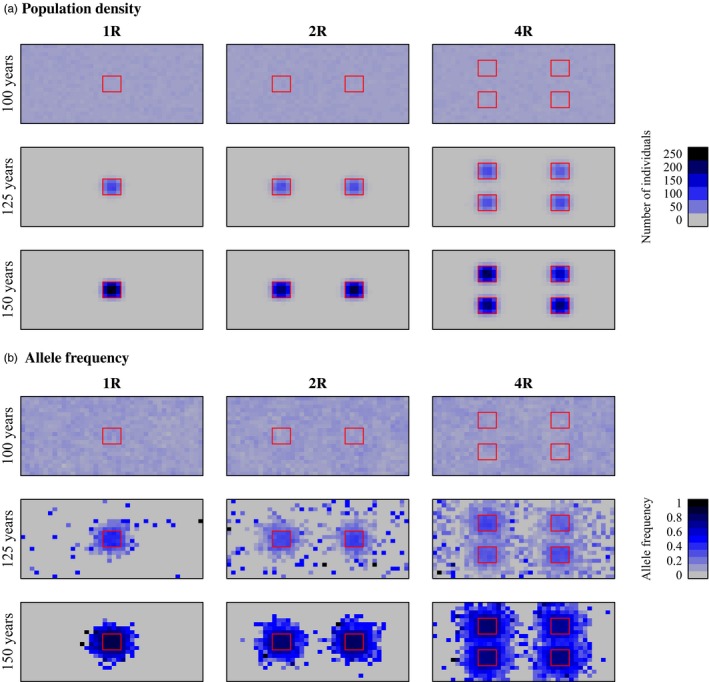
Examples of the change in population density (a: individuals per patch) and evolution of movement rate (b: frequency of the low‐movement‐rate allele) for dogfish. Reserves were established in year 100. Layout and details as in Figure 3. Other parameter values in these simulations are as follows: μ_*AA*_ = 100 NM; μ_*aa*_ = 50 NM;* fished* = 0.225

When fishing mortality was low, within‐reserve population densities of dogfish were significantly higher than population densities without reserves (Table 5, Figure 2c, Fig. S9). When fishing mortality was high, population density continued to decline after reserve establishment, and within‐reserve population density reached zero within 50 years in many instances, especially with little heritable variation in movement rate (Table 5, Figure 2c, Fig. S9). There were, however, significant increases in population density after reserve establishment when movement distances were low and there was sufficient heritable variation in movement distance (i.e., when μ_*AA*_ = 200 NM and μ_*aa*_ = 25 NM, or when μ_*AA*_ = 100 NM and μ_*aa*_ = 50 or 25 NM; Table 5). Marine reserves led to minimal increases in population density in nonreserve grid cells adjacent to marine reserves (Figure 5a).

**Table 5:** Population size and frequency of low‐movement allele for dogfish 50 years after establishment of marine reserves (average over 10 replicate simulations, unless otherwise indicated). Values that are significantly higher than control values (with no reserves), according to Dunnett's test of multiple comparisons to a control, are shown in bold and italic type (*p *< .05). Allele *A* was assumed to act additively throughout (columns in gray indicate cases with no heritability in migration rate)

Fishing mortality	Number of reserves	Movement phenotypes (mean movement distance in NM for the genotypes μ_*AA*_; μ_*aa*_)						
		200; 200	200; 100	200; 50	200; 25	100; 100	100; 50	100; 25
		Within‐reserve population density (individuals per patch)						
Low	(control) 0	478 (*SD *= 4.27)						
	1	***532***	***539***	***549***	***564***	***611***	***626***	***651***
	2	***528***	***537***	***550***	***558***	***608***	***620***	***642***
	4	***527***	***533***	***541***	***554***	***602***	***617***	***635***
High	(control) 0	0 (*SD *= 58.66)						
	1	0^a^	0^a^	9	***327***	3	***167***	**466**
	2	0^a^	0^a^	18	***369***	6	***144***	**472**
	4	0^a^	0^a^	5	***283***	14	***132***	**463**
		Within‐reserve frequency of allele *a*						
Low	(control) 0	0.100 (*SD *= 0.0042)						
	1	0.099	***0.109***	***0.125***	***0.141***	0.099	***0.122***	***0.154***
	2	0.102	***0.112***	***0.126***	***0.140***	0.098	***0.124***	***0.153***
	4	0.098	***0.112***	***0.126***	***0.141***	0.101	***0.124***	***0.149***
High	(control) 0	0.100 (*SD *= 0.0268, unless otherwise noted)						
	1	NA^a^	NA^a^	***1.000*** b	***1.000*** b	0.115	***0.832***	***0.937***
	2	NA^a^	NA^a^	***1.000*** c	***1.000***	0.066	***0.783***	***0.935***
	4	NA^a^	NA^a^	***1.000*** a	***1.000***	0.087	***0.736***	***0.928***

a Population density went to zero in all replicates.

b Average of two replicate simulations (population density went to zero in eight of ten replicates); *SD *= 0.0569.

c Average of five replicate simulations (population density went to zero in five of ten replicates); *SD *= 0.0367.

Average of eight replicate simulations (population density went to zero in two of ten replicates); *SD *= 0.0296.

Average of nine replicate simulations (population density went to zero in one of ten replicates); *SD *= 0.0281.

Consistent with our predictions, there were significant increases in the frequency of the low‐movement‐rate allele following the establishment of marine reserves in all cases where there was heritable variation and population density did not go to zero (Table 5, Figures 2c and 5b, Figs S11 and S12). Movement evolution was more extensive with high fishing mortality than with low fishing mortality, and greater evolutionary changes were seen with higher heritable variation (i.e., when μ_*AA*_ − μ_*aa*_ was large; Table 5, Figure 2c, Figs S11 and S12). These evolutionary reductions in movement rate were generally associated with substantial increases in within‐reserve population sizes over the course of the 50 years following reserve establishment (Figure 2c). The spatial pattern of allele frequency change indicates that the evolution of decreased movement was concentrated in and around reserves (Figure 5b).

### 3.4 Great white sharks

**Figure 6:**
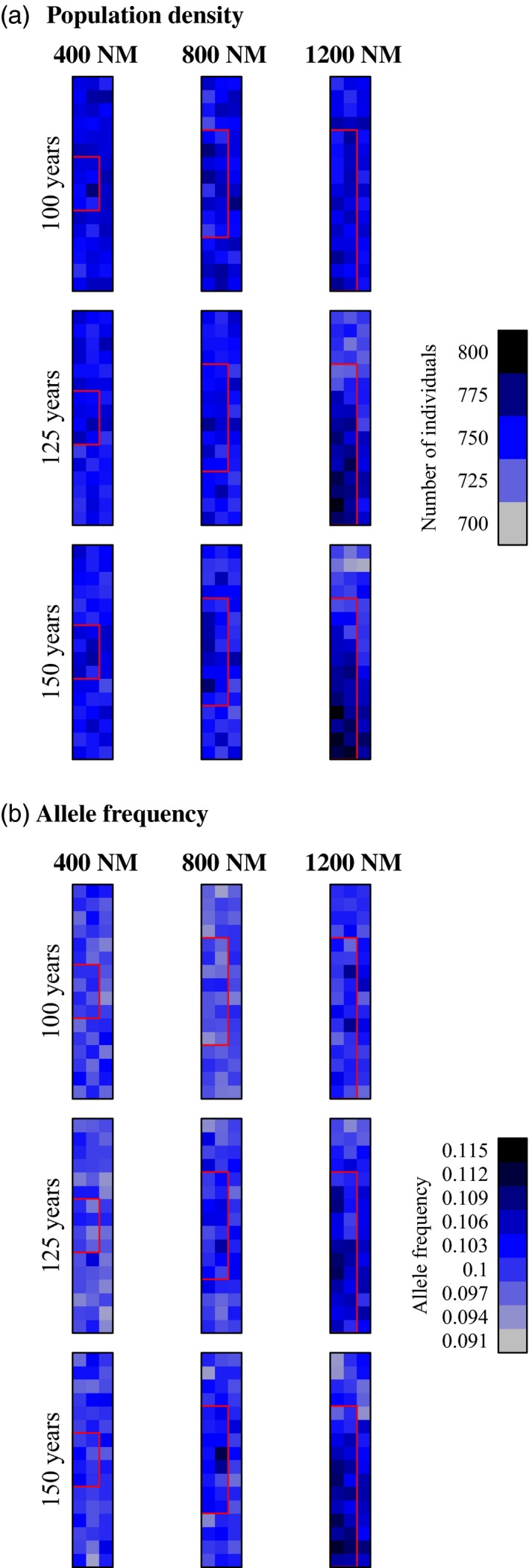
Examples of the change in population density (A: individuals per patch) and evolution of movement rate (B: frequency of the low movement rate allele) for great white sharks across the spawning grounds (feeding grounds not shown). Reserves were established in year 100. Red boxes indicate locations of reserves that span 400 NM (left column), 800 NM (middle column), or 1200 NM (right column) of coastline. Other parameter values in these simulations are as follows: μ_*AA*_ = 600 NM; μ_*aa*_ = 200 NM;* fished* = 0.05

Marine reserves did not cause significant increases in reserve population density except in some scenarios with the largest reserve size and low fishing mortality (Table 6, Figure 2d, Fig. S13). For scenarios with high fishing mortality (*fished* = 0.2), population density continued to decline after reserve establishment and was driven to zero by year 125 (25 years after reserve establishment) in all cases. The establishment of a reserve did not prevent this extinction (Figure 2d, Fig. S14). When fishing pressure was low (*fished* = 0.05), marine reserves caused a slight increase in within‐reserve population density relative to areas outside reserves when the marine reserve was large (Figure 6a). There was a trend of increased within‐reserve population density with decreasing movement distance (Fig. S13).

**Table 6:** Population size and frequency of low‐movement allele for great white sharks 50 years after establishment of marine reserves (average over 10 replicate simulations). Values that are significantly higher than control values (with no reserves), according to Dunnett's test of multiple comparisons to a control, are shown in bold and italic type (*p *< .01). Allele *A* was assumed to act additively throughout (columns in gray indicate cases with no heritability in migration rate)

Fishing mortality	Reserve size	Movement phenotypes (mean movement distance in NM for the genotypes μ_*AA*_; μ_*aa*_)						
		800; 800	800; 600	800; 400	800; 200	600; 600	600; 400	600; 200
		Within‐reserve population density (individuals per patch)						
Low	(control) 0	750 (*SD *= 7.52)						
	400 NM	748	746	754	750	753	752	756
	800 NM	753	748	748	755	754	749	754
	1,200 NM	754	756	757	***759***	***759***	***762***	***767***
High	(control) 0	0 (*SD *= NA)						
	400 NM	0^a^	0^a^	0^a^	0^a^	0^a^	0^a^	0^a^
	800 NM	0^a^	0^a^	0^a^	0^a^	0^a^	0^a^	0^a^
	1,200 NM	0^a^	0^a^	0^a^	0^a^	0^a^	0^a^	0^a^
		Within‐reserve frequency of allele *a*						
Low	(control) 0	0.100 (*SD *= 0.0055)						
	400 NM	0.100	0.099	0.103	0.100	0.098	0.102	0.101
	800 NM	0.102	0.101	0.100	0.098	0.100	0.100	0.103
	1,200 NM	0.101	0.102	0.103	***0.108***	0.102	0.103	**0.106**
High	(control) 0	NA (*SD *= NA)						
	400 NM	NA^a^	NA^a^	NA^a^	NA^a^	NA^a^	NA^a^	NA^a^
	800 NM	NA^a^	NA^a^	NA^a^	NA^a^	NA^a^	NA^a^	NA^a^
	1,200 NM	NA^a^	NA^a^	NA^a^	NA^a^	NA^a^	NA^a^	NA^a^

a Population density went to zero in all replicates.

There was very little evolution of movement rate in our simulations of great white shark. There were significant increases in the frequency of allele *a* in scenarios with low fishing mortality, with the largest reserves, and with the highest heritability (i.e., when reserve size = 1200 NM and μ_*aa*_ = 200 NM; Table 6; Fig. S15). These slight increases in allele frequency were localized within the marine reserve (Figure 6b).

## 4 Discussion

We predicted that the evolution of decreased movement rate following the establishment of marine reserves would lead to higher within‐reserve population size relative to situations with no heritable variation in movement rate. Our results suggest that the evolution of decreased movement rate can augment the efficacy of marine reserves and lead to higher within‐reserve population density, especially for species, such as skipjack tuna, with relatively short generation times. Even when movement rates did not evolve substantially (e.g., given limited time relative to generation length and/or with little heritable variation), marine reserves were an effective tool for the conservation of fish populations when movement rates were low or when reserves were very large (e.g., in the case of great white sharks).

We also predicted that higher fishing mortality (e.g., overfishing) would strengthen selection pressure and lead to faster evolution of movement rate. These effects were seen for every case where overfishing did not lead to extinction. Given the observed association between the evolution of decreased movement rate and increased within‐reserve population size, we contend that marine reserves are an even more effective tool as an insurance policy (e.g., against inaccurate estimates of the maximum sustainable yield) than considered previously, without accounting for evolutionary changes.

Detailed and accurate descriptions of fish population sizes and movement patterns based on tag and recapture data (e.g., Sibert & Hampton, 2003) are crucial to understanding fish stocks, and fisheries scientist have made great strides in identifying the appropriate methods and types of data required for reliable fisheries management (e.g., Punt, Akselrud, & Cronin‐Fine, 2017; Wetzel & Punt, 2015). It is generally the case, however, that the models of fish population dynamics upon which fisheries management is based do not include heritability of life history parameters (such as growth, maturation, or movement rates) and, hence, do not consider the effects of fisheries‐induced evolution (Fraser, 2013; Palkovacs, 2011). Fish stocks do evolve in response to fishing pressure, and there is evidence that this evolution can have significant economic effects (Dunlop, Eikeset, & Stenseth, 2015). Among the studies that investigate the effects of fisheries‐induced evolution, the focus has generally been on the consequences for growth, maturation, and stock recovery. These studies suggest that marine reserves, if properly designed and evaluated on the appropriate time scale, can contribute positively to fishing yield and can reduce the negative impacts of fisheries‐induced evolution, such as small maturation size (Dunlop et al., 2009). If movement rate is heritable, marine reserves may result in locally adapted within‐reserve populations with reduced movement rate that are protected from selection for small maturation size (Miethe et al., 2009).

Importantly, our study suggests that the evolutionary effects of marine reserves are also applicable even to large pelagic fish such as tuna and sharks. In addition, we show that increasing the size or number of marine reserves enhances their overall efficacy for conservation purposes and that the efficacy is augmented by the evolution of movement rate regardless of reserve size or number. Our finding regarding the benefits of multiple reserves is in alignment with previous analyses that suggest the need for multiple closely spaced reserves to maintain connectivity and preserve populations of fishes, especially in light of increasing sea surface temperatures (Andrello, Jacobi, Manel, Thuiller, & Mouillot, 2015; Gerber, Mancha‐Cisneros, O'Connor, & Selig, 2014). Overall, we contend that movement rate, like many traits, is more likely to be heritable than not and consideration of the evolutionary effects of marine reserves and fishing should be incorporated into fisheries management and spatial conservation planning.

In our simulations, the evolution of decreased movement rate within reserves was likely restricted by gene flow from outside of the reserves. Although movement rate is neutral outside of the reserves, and there is, therefore, no selection favouring the high movement rate allele outside the reserves, the strength of selection, averaged across the whole population, will still be diminished by gene flow. Hence, in order for adaptation to proceed rapidly in the reserves, selection must overwhelm the effect of gene flow from the surrounding populations; otherwise, gene swamping will occur (Gomulkiewicz & Holt, 1995; Gomulkiewicz, Holt, & Barfield, 1999; Holt & Gomulkiewicz, 1997). Selection was least effective when movement rates were so high that swamping was substantial and few fish would remain within the reserve, especially if genetic differences in movement rate were not substantial (e.g., compare thickest to thinnest arrows in Figure 2). As a consequence, species with lower movement distances (e.g., dogfish) tended to exhibit greater evolutionary responses over the 50‐year time window than highly migratory species (e.g., bluefin). We did not include any larval drift (between cells) in our models, and the amount of swamping may, therefore, be underestimated in our study. An analysis of the effect of the distance and direction of larval drift on the evolution of movement rates is a potential avenue for future research on marine reserves.

Whereas the focus of our research was on the conservation implications of marine reserves, we acknowledge the potentially important benefit of marine reserves to fisheries from spillover—increases in catch adjacent to reserves (Roberts, Bohnsack, Gell, Hawkins, & Goodridge, 2001). This is an interesting topic in the context of evolving movement rate. We have shown that high heritability of movement rate results in locally adapted philopatric populations with high population densities within reserves. Whereas increased population density within reserves should result in more spillover (Halpern & Warner, 2002; Roberts et al., 2001), reduced movement should result in less per‐capita spillover. The net effect (of increased density and decreased movement) on spillover is unclear, and a quantification of these effects on catch rates adjacent to marine reserves would constitute another interesting avenue for future research.

We did not model any particular fitness benefit associated with high movement distance and did not account for the advantages of movement to the survival and fitness of pelagic fish. One could hypothesize the existence of several such benefits. For example, high movement distances might have evolved due to a need to exploit spatially and/or temporally fluctuating resources. We assumed resources were fixed and homogenous across the simulation grid. In addition, we did not model any particular fitness cost associated with low movement distance, but such costs might exist. For example, low movement distances may cause negative density effects on populations (e.g., increased adult mortality or lower fecundity), as more stationary adults would tend to accumulate in particular locations (such as within reserves). We assumed that only fry survival was density dependent. Future work on marine reserves could investigate the effects of additional density dependence (e.g., density‐dependent adult survival or movement distance), as well as the effects of fluctuating resources. Although such factors would undoubtedly have a quantitative effect on the results, the qualitative impact of the strong selection on migration due to high fishing pressures is likely to augment the effectiveness of marine reserves through the evolution of movement rate across a broader range of assumptions than considered here.

While our simulations are highly simplified representations, our study makes two important points that are generalizable. First, whereas the effectiveness of marine reserves may vary for different species of pelagic fishes depending on their life history characteristics, this effectiveness is most certainly underestimated if the potential for evolution of movement rate is neglected. We note that, in our simplified model of movement evolution based on a one‐locus two‐allele system, heritability was relatively low (maximum heritability was 0.07), and hence our estimates of the time scale of evolution are conservative relative to cases with higher heritability (e.g., when multiple genes contribute to movement rate). Second, if evolution of movement rate occurs due to fishing mortality outside of reserves, the rate of evolutionary change will be highest when mortality is highest (i.e., when selection is strongest), and marine reserves can therefore act as an important insurance policy against target‐based management gone awry. These results provide support for the use of large marine reserves as a tool for the conservation of large pelagic fishes. Our model results could potentially be validated by studying changes in movement distance and population size in fish populations in and around recently established no‐take marine reserves, such as the Phoenix Islands Protected Area (established in 2015) and the Pacific Remote Islands Marine National Monument (established in 2009 and expanded in 2014).

## Data archiving statement

The simulated data sets, and simulation code, used in this work have been uploaded to the Dryad Digital Repository: https://doi.org/10.5061/dryad.jm40h.
