# Supplementary material for: Evolution of movement rate increases the effectiveness of marine reserves for the conservation of pelagic fishes
Source: Evol Appl. 2017 Mar 15;10(5):444–61. doi: 10.1111/eva.12460 (PMC5427674; doi:10.1111/eva.12460)
Supplement: Supplementary file 1 [file EVA-10-444-s001.docx]

Supplementary Figures (Figures S1 to S16)

**Evolution of movement rate increases the effectiveness of large marine reserves for the conservation of pelagic fishes**

**Figure S1.** Within-reserve population densities (individuals per patch) of skipjack tuna with low fishing mortality. The top, middle, and bottom rows (across both panels) show the outcomes of establishing one, two, or four reserves, respectively. LEFT PANELS: mean population density (individuals per patch) within reserves across all replicates (n = 10) from years 100 to 150. Marine reserves were established in year 100, and coloured lines show population densities after reserve establishment. Black lines show the population trajectory when no reserves were established. Colours correspond to simulations with different combinations of values for the *μ_AA_* and *μ_aa_* parameters, as indicated in the right panels. RIGHT PANELS: box plots show median population densities within reserves at 150 years (50 after reserve establishment), 1^st^ and 3^rd^ quantiles (box margins and whiskers), and outliers (points) from ten replicate simulations. Population size at year 150 with no reserves (0R) is shown at the left of the plots.

**Figure S2.** Within-reserve population densities (individuals per patch) of skipjack tuna with high fishing mortality. Details as in Figure S1.

**Figure S3.** Evolution of movement rate in skipjack tuna simulations with low fishing mortality. Layout and colours as in Figure S1, except showing mean frequencies of the *a* (low movement rate) allele within reserve patches across all replicates (n = 10) from years 100 to 150. If populations went extinct in > 8 replicates, boxplots were replaced by “NA”.

**Figure S4.** Evolution of movement rate in skipjack tuna simulations with high fishing mortality. Layout and colours as in Figure S3.

**Figure S5.** Within-reserve population densities of Bluefin tuna with low fishing mortality. Layout and colours as in Figure S1.

**Figure S6.** Within-reserve population densities of Bluefin tuna with high fishing mortality. Layout and colours as in Figure S1.

**Figure S7.** Evolution of movement rate in bluefin tuna simulations with low fishing mortality. Layout and details as in Figure S3.

**Figure S8.** Evolution of movement rate in Bluefin tuna simulations with high fishing mortality. Layout and details as in Figure S3.

**Figure S9.** Within-reserve population densities of dogfish with low fishing mortality. Layout and colours as in Figure S1.

**Figure S10.** Within-reserve population densities of dogfish with high fishing mortality. Layout and colours as in Figure S1.

**Figure S11.** Evolution of movement rate in dogfish simulations low fishing pressure. Layout and details as in Figure S3.

**Figure S12.** Evolution of movement rate in dogfish simulations with high fishing mortality. Layout and details as in Figure S3.

**Figure S13.** Within-reserve population densities of great white sharks with low fishing mortality. Layout and colours as in Figure S1.

**Figure S14.** Within-reserve population densities of great white sharks with high fishing mortality. Layout and colours as in Figure S1.

**Figure S15.** Evolution of movement rate in great white shark simulations with low fishing mortality. Layout and details as in Figure S3.

**Figure S16.** Evolution of movement rate in great white shark simulations with high fishing mortality. Layout and details as in Figure S3.
